# Editorial: Biomaterial applications in soft tissue engineering and replacement

**DOI:** 10.3389/fbioe.2023.1227021

**Published:** 2023-05-30

**Authors:** István Hornyák, Angéla Jedlovszky-Hajdú, Seda Kehr

**Affiliations:** ^1^ Institute of Translational Medicine, Semmelweis University, Budapest, Hungary; ^2^ Laboratory of Nanochemistry, Department of Biophysics and Radiation Biology, Semmelweis University, Budapest, Hungary; ^3^ Izmir Institute of Technology, Department of Chemistry, Urla, Türkiye

**Keywords:** material science and engineering, biomaterial, biocompatibility, cell vialbility, *in vivo* application

The research related to the application of biomaterials encompasses a large area within the field of tissue engineering and regenerative medicine (TERM), and this Research Topic was dedicated to the versatile possibilities in the use of biomaterials. The sum of 10 manuscripts were submitted to this Research Topic and six were selected for this Research Topic with the contribution of 35 authors, Four of the accepted manuscripts were original research articles and two were review articles.

One of the most interesting aspects of biomaterials is that we are able to investigate the whole life-cycle of the chosen material, as a probable first step, there is the modeling and material science. Generally, when we try to develop a new material, the surface and composition can be evaluated using various spectroscopic methods, e.g., Fourier transform infrared spectroscopy (FTIR), X-ray photoelectron spectroscopy (XPS), and microscopic methods, e.g., digital microscope, scanning electron microscopy (SEM), or fluorescent microscopy. These methods need to be chosen in accordance with the type of starting materials and manufacturing, which can be another aspect to divide biomaterials into the appropriate categories, as metal-based materials are generally not suitable for FTIR, fluorescent microscopy or generally in swelling or enzymatic decomposition related characterization, but their pathway or elimination can be followed in the living system for example, with magnetic resonance imaging (MRI), positron emission tomography (PET), computed tomography (CT). The manufacturing methods can mainly be divided into the following: phase separation (precipitation), rapid prototyping, supercritical fluid technology, porogen leaching, electrospinning, 3D printing, freeze drying, centrifugal casting, templating, and micro patterning (Collins and Birkinshaw, 2013; Tóth et al., 2023). However, generally the main requirement towards a biomaterial is to improve tissue regeneration and to enable to create an environment that supports the attachment, proliferation, migration and differentiation of cells (Juriga et al., 2022; Zhang et al.).

One of the biomaterials that have been in use for the longest time are metals, thus, it is safe to say that this type of materials made it through the test of time, however, we can still witness developmental directions, both in the manufacturing and in the treatment of metallic biomaterials. Regarding the manufacturing, the traditional method is the casting of metals, but 3D printing of metals is quickly gaining interest, however, as the regulation of 3D printed medical devices is not clear yet, thus still casted materials are applied in medical devices (Burnard et al, 2020). The main treatment options are heat treatment to improve the characteristics, leaching, which is still in the R&D phase not application, and sterilization, which is generally required for metals to be applied as a medical device. However, 3D printing of metals is the most trending method, an example is how the surface treatment of 3D printed titanium can enhance cell attachment (Kulcsár et al.).

The other large family of biomaterials are organic components, within the field of TERM, these especially mean biodegradable materials with sufficient mechanical integrity for the length of the application and then are ultimately eliminated and the area of the treatment is remodeled. The most frequent research is the investigation of scaffolds, usually in an advanced *in vitro* and/or *in vivo* experimental setup that can lead to advanced biomaterial applications. These investigations usually start with gathering proof that the used material is in fact a biomaterial, without any toxicity, usually based on *in vitro* experiments. The potential effect may be evaluated with the use of cell viability assays, cell migration assays, 3D imaging with dyes, and if a special regenerative function is tested, then with the use of gene expression measurements. Among these trending biomaterials are hyaluronic acid, collagen, polycaprolactone (PCL), poly lactic acid (PLLA) and their derivatives, which have already been used in medical devices, thus these are generally considered to be safe to use even as a permanent soft tissue implant (Hinsenkamp et al., 2021; Hinsenkamp et al., 2022b). These investigations are especially useful from the viewpoint of application, as there are materials that do well under *in vitro* circumstances, however the *in vivo* results suggest that the *in vivo* application may not be realistic, alginate is typically such a material, and is often debated alone or in combination with other biomolecules (Zhang et al.).

Organic/inorganic composite biomaterials, in which organic polymers/hydrogels interact with inorganic nanoparticles, are class of advanced biomaterials. Such composite systems offer advanced properties due to the synergistic combination of soft, flexible polymers with hard inorganic nanoparticles with unique optical, mechanical, and electrical properties. This type of biomaterials is responsive to stimuli, injectable and biodegradable, making it ideal for 3D artificial biomaterials, especially for applications in tissue engineering, wound healing and controlled drug delivery (Motealleh and Kehr, 2017).

The transition from an *in vitro* setup to and *in vivo* model is one of the most challenging parts in the life-cycle of biomaterial application related research and development, as this is the step where limited variable that are present in the material science related research and the *in vitro* limitations are no longer present. Thus, in this step it becomes important, that the materials can be manufactured in a reproducible manner, and they are implanted for both a shorter and for a longer term, e.g., 4 and 12 weeks (Hinsenkamp et al., 2022b). In this step the toxicology data from the *in vitro* part can be a good indication, but the moving sterical surroundings, and the enzymatic interventions may present completely different requirements compared to the *in vitro* circumstances.

The usability of biomaterials can also be approached from the viewpoint of *in vivo* functionality, and one of the challenging fields is the replacement of tendons, as these are not densely populated by cells, and alignment is a must have, as well as improved mechanical strength, which limits the applicable materials and the used method. As an example, the material which may have enough mechanical strength is PCL, and the method, which can allow the alignment of the fibers mimicking the structure of tendons (Adeoye et al.).

When we arrived to the conclusion with our biomaterial that it can considered to be safe and fulfills the requirements of our intended use in the *in vivo* setup, we can start to develop a specific biological activity, in order to achieve a targeted therapeutic effect. These can be reached with the modification of the surface of the biomaterial, e.g., with the coating of the material with the biologically active materials enabled by simple secondary bonds, or covalent bonds, even on a micro or nano scale (Abesekara and Chau). These methods may lead to functionalized biomaterials, which can now act as controlled or sustained delivery system for peptides or growth factors and can interact with pre-determined cell types (Tang et al.) or may be suitable to enhance the regeneration of a pre-defined type of injury (Zhang et al.).

Regarding the life-cycle of the biomaterial, with the use of coating and functionalization leading to a new cycle due to the new features, the release kinetics and degradation profile has to be established again, as well as a new set of *in vitro* and *in vivo* toxicology and biocompatibility measurements, with the main difference that while the starting biomaterial may be a medical device, however, with the addition of biologically active materials the biomaterial will now certainly need to be regulated as a drug, in order to be applied, which increases the cost and the risk of the actual therapeutic application. The early approach of imagining the biomaterial as a therapeutically applicable product is strongly suggested for the R&D colleagues throughout the field of TERM, as it may help with the evaluation of the optimal outcome of the potential biomaterial application including intellectual property, publication, regulatory and actual product development, as in an best-case scenario at the end of the life-cycle of a biomaterial development is an actual product that can reach the market (Hinsenkamp et al., 2022a).
